# The anaerobic degradation of gaseous, nonmethane alkanes — From in situ processes to microorganisms

**DOI:** 10.1016/j.csbj.2015.03.002

**Published:** 2015-03-19

**Authors:** Florin Musat

**Affiliations:** Helmholtz Centre for Environmental Research — UFZ, Permoserstr. 15, 04318 Leipzig, Germany

**Keywords:** Gaseous alkanes, Microbial degradation, Anaerobic, Marine, Sediments, Sulfate

## Abstract

The short chain, gaseous alkanes ethane, propane, *n*- and *iso*-butane are released in significant amounts into the atmosphere, where they contribute to tropospheric chemistry and ozone formation. Biodegradation of gaseous alkanes by aerobic microorganisms, mostly bacteria and fungi isolated from terrestrial environments, has been known for several decades. The first indications for short chain alkane anaerobic degradation were provided by geochemical studies of deep-sea environments around hydrocarbon seeps, and included the uncoupling of the sulfate-reduction and anaerobic oxidation of methane rates, the consumption of gaseous alkanes in anoxic sediments, or the enrichment in ^13^C of gases in interstitial water vs. the source gas. Microorganisms able to degrade gaseous alkanes were recently obtained from deep-sea and terrestrial sediments around hydrocarbon seeps. Up to date, only sulfate-reducing pure or enriched cultures with ethane, propane and *n*-butane have been reported. The only pure culture presently available, strain BuS5, is affiliated to the *Desulfosarcina*–*Desulfococcus* cluster of the *Deltaproteobacteria*. Other phylotypes involved in gaseous alkane degradation have been identified based on stable-isotope labeling and whole-cell hybridization. Under anoxic conditions, propane and *n*-butane are activated similar to the higher alkanes, by homolytic cleavage of the C—H bond of a subterminal carbon atom, and addition of the ensuing radical to fumarate, yielding methylalkylsuccinates. An additional mechanism of activation at the terminal carbon atoms was demonstrated for propane, which could in principle be employed also for the activation of ethane.

## 1 Introduction

The short chain, nonmethane gaseous alkanes ethane, propane, *n*-butane and *iso*-butane are major constituents of natural gas. The gaseous alkanes are also abundant in crude oil reservoir gas caps, and are found in smaller amounts dissolved in crude oil [1]. They are generally formed through the abiotic, thermal decomposition of fossil organic matter, but are also produced biologically, as shown recently for ethane and propane (e.g. [2,3]). Ethane and propane are also produced in significant amounts by biomass burning, leading to their direct release into the atmosphere [4]. The gaseous alkanes reach the biosphere through vertical migration of gas and oil from deep seated reservoirs, along fractures in the crust caused by plate tectonics, or as a result of anthropogenic activities. Migration through the sediment takes place as free gas or as dissolved constituents of geothermal fluids. The short-chain alkanes are released into marine and terrestrial environments via microseepage, which accounts for the bulk emissions, onshore and marine seeps, and mud volcanoes [5]. Seepage in environments characterized by low temperatures and elevated pressures, such as the deep seafloor and permafrost regions, leads to formation of type II gas hydrates [6]. Although methane is still the most abundant hydrocarbon, gas hydrates in the Gulf of Mexico and Caspian Sea contain over 30% C_2_–C_4_ alkanes [7]. The short-chain alkanes are eventually released into the atmosphere, contributing to the tropospheric chemistry as important precursors for the formation of ozone and organic aerosols [8]. Global atmospheric emissions are estimated at 9.2–9.6 Tg yr^− 1^ for ethane, 9.6–10.5 Tg yr^− 1^ propane, 10 Tg yr^− 1^ *n*-butane and 4.2 Tg yr^− 1^ *iso*-butane [4,5]. The amounts reaching the atmosphere may be severely limited by their biodegradation by aerobic and anaerobic microorganisms in marine sediments or in the water column, and in terrestrial environments.

## 2 Aerobic degradation of short chain alkanes

Microorganisms able to grow with gaseous alkanes as substrates under aerobic conditions are known since several decades. Among the first bacterial pure cultures reported were strains assigned to *Mycobacterium* spp., isolated or otherwise able to grow with ethane, propane or *n*-butane (e.g. [9,10]). Most of the strains isolated so far with gaseous alkanes are affiliated with the high G + C Gram-positive bacteria, e.g. *Arthrobacter*, *Corynebacterium*, *Mycobacterium*, *Nocardia* and *Rhodococcus* [11]. Comparatively, a smaller number of strains affiliated with the Proteobacteria (*Gamma* and *Betaproteobacteria*) have been reported [11]. In addition to bacteria, cultures of fungi able to degrade gaseous alkanes have been isolated. The diversity of aerobic, gaseous alkane degrading bacteria and fungi has been reviewed recently [11], and it will not make the subject of the present review. However, it is worth noting that the vast majority of microbial strains reported so far have been isolated from soil, or otherwise terrestrial environments (see [11] and the references therein). Marine bacteria able to degrade C_2_–C_4_ alkanes have been seldom reported, despite the fact that the oceans receive large inputs of hydrocarbons from natural seepage or anthropogenic activities. Instead, filamentous fungi isolated with crude oil from coastal marine sediments have been found to degrade also gaseous alkanes [12]. Marine bacteria involved in the aerobic degradation of ethane and propane have been recently identified by incubation of surface sediment from a hydrocarbon seep with ^13^C-labeled substrates, followed by DNA stable isotope probing [13]. Ethane was apparently degraded by members of *Methylococcaceae* (*Gammaproteobacteria*), and propane by members of an unclassified group of *Gammaproteobacteria* [13]. Among the strains most closely related to the propane-degrading phylotypes were obligate hydrocarbon degraders of the genera *Marinobacter* and *Alcanivorax*, isolated from marine environments [13,14]. The degradation of gaseous alkanes in marine environments received more attention in the recent years. Analyses of stable isotope fractionation factors showed that gaseous alkanes were biologically degraded under oxic conditions in incubations with marine sediments from hydrocarbon seeps [15], and in the water column above mud volcanoes in the Nile deep-sea fan [16]. A preferential oxidation of propane and *n*-butane over ethane was observed [15]. Following the Deepwater Horizon event large amounts of natural gas were emitted in deep waters of the Gulf of Mexico [17]. The gas formed plume structures, with the highest concentrations at depths greater than 800 m. Ethane and propane were degraded in fresh plumes, accounting for up to 70% of the observed oxygen depletion [17].

Under aerobic conditions, the gaseous alkanes are typically activated at the terminal carbon atom by reactive oxygen species derived from molecular oxygen, yielding primary alcohols [11]. For propane, a second route of activation at the secondary carbon atom, yielding 2-propanol, has been described [11]. The activation reaction is catalyzed by monooxygenases (see [18], for an overview of the activation mechanism). Most of the bacterial gaseous alkane monooxygenases are soluble di-iron monooxygenases (e.g. [11,19,20]). A copper-containing monooxygenase, similar with the particulate methane monooxygenase and ammonia monooxygenase, was proposed for a strain of *Nocardioides* [21]. Activation of gaseous alkanes in fungi was proposed to be carried out by cytochrome P-450 monooxygenases [22]. The primary alcohols are further oxidized to aldehydes and fatty acids, which are channeled into central metabolic pathways, and typically oxidized completely to CO_2_ [11]. In case of propane activation at the secondary carbon atom, the formed 2-propanol is oxidized to propanone (acetone), and further to hydroxypropanone for which several routes of degradation have been proposed, including oxidation to acetate or to pyruvate [11]. Degradation of *iso*-butane has been less frequently treated in the literature devoted to gaseous alkane degradation. *Iso*-butane can be activated at a primary carbon atom, yielding isobutanol, which can be further degraded via *iso*-butylaldehyde and *iso*-butyrate [23]. Alternatively, *iso*-butane could be activated at the tertiary carbon atom, to *tert*-butanol. Subsequent oxidation may lead to 2-hydroxyisobutyrate. Degradation of the 2-hydroxyisobutyrate, a compound containing a tertiary carbon atom, has been shown to proceed via a carbon skeleton rearrangement catalyzed by a mutase [24].

## 3 Anaerobic degradation of short chain alkanes — evidence from in situ studies

The anaerobic degradation of short-chain alkanes was for the first time suggested by geochemical studies of deep-sea marine sediments around hydrocarbon seeps. These studies preceded the isolation and cultivation of the first anaerobic microorganisms able to degrade gaseous alkanes. For consistency, all studies addressing the in situ degradation will be summarized here. In environments affected by hydrocarbon seepage, the sulfate reduction rates (SRRs), which were usually attributed to the anaerobic oxidation of methane (AOM), were much higher than the rates of AOM. In Gulf of Mexico sediments around gas hydrates, the SRRs were two to three orders of magnitude higher than the AOM rates [25,26]. This difference was even more pronounced in Gulf of Mexico sediments associated with type II gas hydrates, which contain high concentrations of C_2_–C_5_ alkanes [27]. Other sites with high ratios of SR rates versus AOM rates and with confirmed presence of short chain alkanes include mud volcanoes in the Gulf of Cadiz [28,29], mud volcanoes in the Eastern Mediterranean Sea [30,31], crude oil and gas cold-seeps in the Gulf of Mexico [32], the Chapopote asphalt volcano [32], Mississippi Canyon sediments [33], and hydrothermal sediments from the Guaymas Basin [34]. These rate measurements have been recently summarized with calculation of a global median ratio of about 10:1 for SR vs. AOM rates [35]. The discrepancy between SR and AOM rates was generally attributed to the oxidation of hydrocarbons other than methane, including short-chain alkanes, by sulfate-reducing microorganisms (e.g. [32,35]).

The anaerobic oxidation of gaseous alkanes has been proposed to contribute to the formation of dry gas caps (enriched in methane due to the degradation of C_2_–C_5_ alkanes) associated with biodegraded oil [36], and to the formation of carbonate precipitates around gas hydrates in the Gulf of Mexico [37]. A decrease in the concentrations of gaseous alkanes within the sulfate–methane transition zone at mud volcanoes in the Gulf of Cadiz could also be due to degradation of these compounds under anoxic conditions [28]. Measurements of isotopic composition of gaseous alkanes in marine sediments associated with gas hydrates in the Gulf of Mexico showed that propane and *n*-butane were enriched in ^13^C in sediment interstitial water relative to vent gas and to gas in hydrates [38]. This was proposed to be due to anaerobic microbial oxidation. Ethane and *iso*-butane, enriched to a lesser extent in ^13^C relative to the source gas, appeared relatively stable to microbial degradation [38]. Stable isotope fractionation analyses also showed that propane and *n*-butane were preferentially degraded versus ethane and *iso*-butane in anoxic sediments of deep-sea mud volcanoes in the Mediterranean Sea [16].

The anaerobic degradation of gaseous alkanes in the environment was also indicated by the finding of metabolites typical for the biochemical activation under anoxic conditions. Ethyl- and propylsuccinates, indicative of anaerobic ethane and propane degradation, were detected in samples from crude oil contaminated aquifers [39], in oilfields [40], and coal beds [41]. Alkylsuccinates suggestive of anaerobic degradation of ethane, propane and *n*-butane were also detected in samples from oil processing facilities [42]. This indicated that the gas repeatedly injected into reservoir in order to increase oil recovery may have led to the enrichment of microorganisms able to degrade gaseous alkanes.

In addition to direct in situ observations, anaerobic degradation of gaseous alkanes has been also determined in laboratory incubations with sediment samples. The anaerobic degradation of propane was demonstrated in incubations of fresh sediment samples from marine hydrocarbon seeps with ^13^C-propane under sulfidogenic conditions [43]. Preferential oxidation of propane and *n*-butane in Gulf of Mexico sediments was recently demonstrated by stable isotope analyses in incubations of hydrocarbon-rich sediment samples with individual alkanes [44]. Also, batch sediment incubations with sediments from hydrothermal vent systems at Middle Valley, Juan de Fuca Ridge showed that ethane, propane and *n*-butane were degraded under sulfate-reducing conditions over a broad range of temperatures (25°, 55° and 75 °C) [45]. Overall, the in situ studies and ex situ incubations point out that anaerobic microorganisms able to degrade SCA are relatively widespread and physiologically diverse, at least with respect to the range of temperatures at which SCA degradation has been observed.

## 4 Microorganisms degrading SCA under anoxic conditions

The first anaerobic microorganisms able to degrade short-chain alkanes were enriched and isolated from marine hydrocarbon seep sediments [46]. Very slow ethane-dependent sulfate reduction was shown at 12 °C with an enrichment culture from Gulf of Mexico sediments. Cold-adapted (12 °C), sediment-free enrichment cultures were obtained with propane and *n*-butane as substrates from Gulf of Mexico sediments, and with *n*-butane from Hydrate Ridge sediments [46,47]. Mesophilic (28 °C) and thermophilic (60 °C) enrichment cultures with propane and *n*-butane were obtained from Guaymas Basin sediments; from the mesophilic enrichment culture with *n*-butane a pure culture, strain BuS5, was isolated [46]. Substrate tests showed that strain BuS5 was able to use only propane and *n*-butane, but not shorter (ethane, methane), or longer chain alkanes (e.g. pentane) [46]. This narrow hydrocarbon substrate range, i.e. the ability to degrade only propane and *n*-butane, suggesting a restricted environmental distribution of these microorganisms, was later shown also for cold-adapted enrichment cultures from the Gulf of Mexico and Hydrate Ridge [47]. Mesophilic, propane-degrading sulfate-reducing microorganisms were also enriched from a low-temperature, sulfidic hydrocarbon seep [48]. To date, no pure or enriched culture able to oxidize *iso*-butane under anoxic conditions has been reported.

**Fig. 1:**
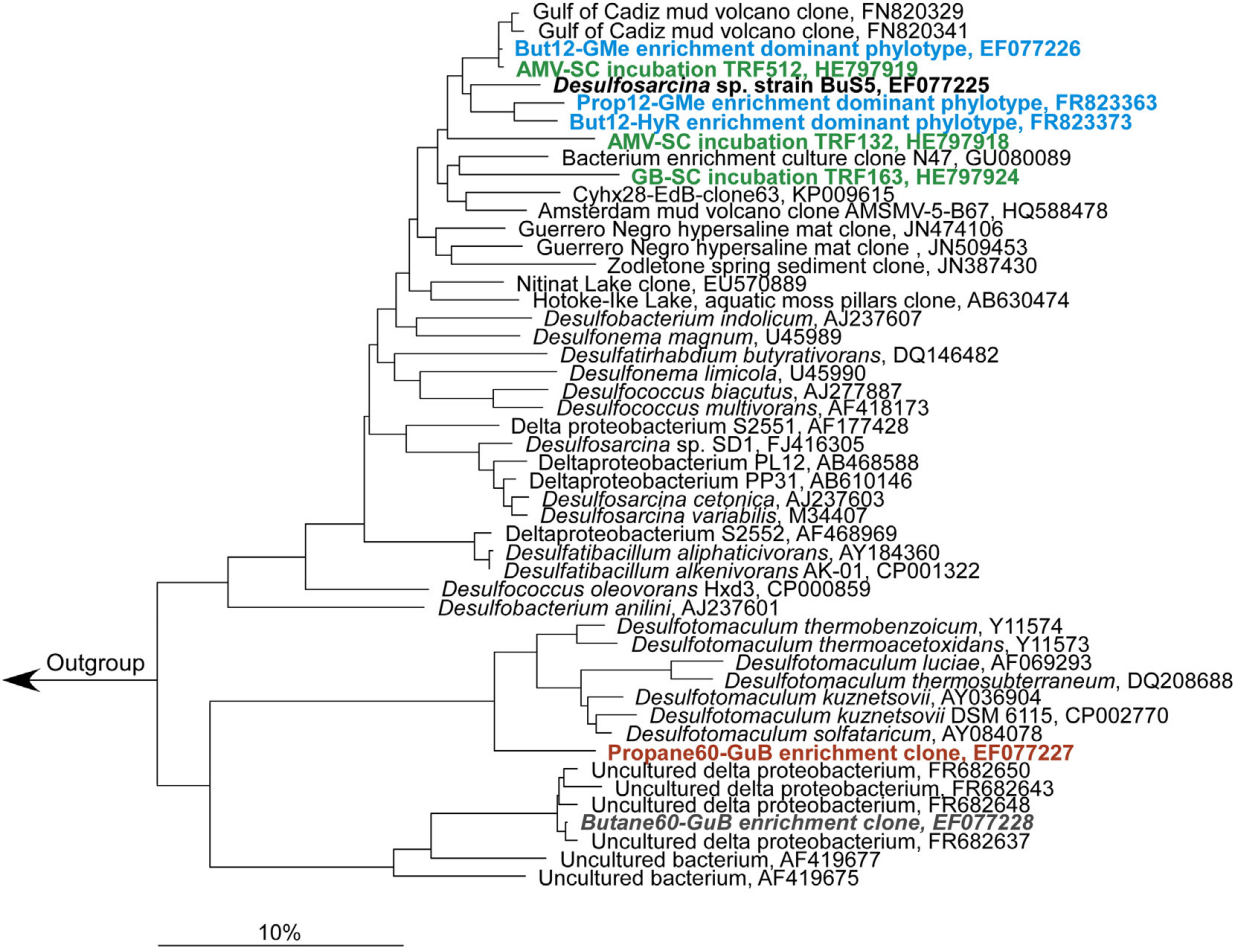
Phylogenetic reconstruction of anaerobic short-chain alkane degraders. Strain BuS5 (bold), the only pure culture isolated so far able to degrade propane and *n*-butane, is affiliated with the *Desulfosarcina*–*Desulfococcus* group of the *Deltaproteobacteria*. Dominant phylotypes involved in propane or *n*-butane degradation in enrichment cultures (blue) were identified based on labeling with ^13^C-substrates followed by chemical imaging via nanoSIMS analysis. Additional phylotypes involved in SCA degradation were identified by stable isotope probing in incubations with marine sediments (green). A phylotype related to *Desulfotomaculum* spp. (red) was proposed to be responsible for propane degradation based on its abundance in a sulfate-reducing, thermophilic enrichment culture. The tree was reconstructed in ARB by maximum likelihood, using only nearly full-length sequences (> 1400 bp). Partial sequences were added by parsimony to the calculated tree. Scale bar indicates an estimated 10% sequence divergence.

All anaerobic SCA degraders identified so far are sulfate-reducing bacteria. Strain BuS5, which to date is the only pure culture available able to degrade SCA under strictly anoxic conditions, is phylogenetically affiliated with the *Desulfosarcina*–*Desulfococcus* cluster of the *Deltaproteobacteria* [46] (Fig. 1). Strain BuS5 is closely related to other hydrocarbon-degrading bacteria, including degraders of *n*-alkanes > C_6_ and of aromatic hydrocarbons [49]. Using incubations with ^13^C-labeled substrates followed by probing with fluorescently-labeled oligonucleotide probes and chemical imaging via nanoSIMS analysis, propane and butane-degrading sulfate-reducing bacteria were identified in cold-adapted enrichment cultures from the Gulf of Mexico and Hydrate Ridge [47]. These microorganisms were closely related to strain BuS5, forming an apparent phylogenetic cluster of SCA degraders (Fig. 1). The phylogenetic diversity of potential SCA degrading microorganisms was also investigated in batch incubations of Gulf of Mexico sediments with gaseous alkanes [44]. The sequences retrieved were closely related to the BuS5 group, although a relatively high diversity of Deltaproteobacterial sequences was retrieved from the incubations with ethane [44]. In a recent study, incubations with ^13^C-labeled substrates followed by stable isotope probing showed that microorganisms affiliated with the strain BuS5 cluster were involved in the in situ degradation of *n*-butane at hydrocarbon seeps in the Eastern Mediterranean Sea and Guaymas Basin [50] (Fig. 1). In this latter study, a relatively high diversity of potential SCA degraders in marine sediments around hydrocarbon seeps was identified.

Molecular biology methods were also used to describe the microbial community structure of a terrestrial propane-degrading enrichment culture [48]. A relatively high phylogenetic diversity was found, with several phylotypes affiliated with the *Deltaproteobacteria*. Although the microorganisms involved in propane degradation were not directly identified, it is worth noting that none of the phylotypes in this enrichment culture was closely related with the strain BuS5 cluster [48]. This suggests that, in addition to the marine SCA cluster, different phylogenetic groups may be responsible for the anaerobic SCA degradation in low temperature terrestrial environments.

Hybridizations with sequence-specific, 16S rRNA gene-targeted oligonucleotide probes showed that a sulfate-reducing, thermophilic enrichment culture with propane was largely dominated by a phylotype affiliated with *Desulfotomaculum* spp. [46] (Fig. 1). Due to its high abundance, of over 90% of the total cell number, this phylotype is most likely responsible for propane degradation. Construction and analysis of a 16S rRNA gene library from a thermophilic (60 °C) enrichment culture with butane yielded a single phylotype (Butane60-GuB) forming a novel phylogenetic cluster within the *Deltaproteobacteria* [46]. However, the abundance of this phylotype relative to the total cell number was not determined, and its role in *n*-butane degradation remains speculative. A high diversity of sequences closely related to the Butane60-GuB phylotype was recently retrieved from thermophilic (55 °C) enrichment cultures with ethane, propane and *n*-butane, and have been suggested to represent a group of thermophilic SCA degraders [45]. Nevertheless, future studies including for example cultivation (e.g. isolation of pure cultures) or stable isotope labeling coupled with phylogenetic identification (e.g. SIP or chemical imaging via nanoSIMS), are needed to elucidate the role of this phylogenetic cluster in thermophilic SCA degradation.

## 5 Biochemistry of SCA degradation under anoxic conditions

The solubility in water of the gaseous alkanes is relatively high compared with that of higher alkanes, of 2.13 mmol l^− 1^ (ethane), 1.76 mmol l^− 1^ (propane), 1.46 mmol l^− 1^ (*n*-butane), and 0.94 mmol l^− 1^ (*iso*-butane) at 20 °C (calculated from Ostwald coefficient, according to [51]; see also [52] for a comparison of *n*-alkane solubility depending on the chain length). In the absence of a proven active uptake mechanism, the dissolved alkanes are assumed to diffuse freely into the cells, via partitioning into cellular membranes. Anaerobic microorganisms make use of fundamentally different co-substrates for the activation of hydrocarbons. The most well described mechanism of activation is the radical-catalyzed homolytic cleavage of the C—H bond, followed by the carbon–carbon addition of the alkyl radical to fumarate, which acts as a co-substrate. Hydrocarbon activation by addition to fumarate was initially described for the anaerobic degradation of toluene, where it yields benzylsuccinate [53,54]. The reaction is catalyzed by benzylsuccinate synthase [55]. Benzylsuccinate synthase and its homologs are glycyl radical enzymes of the pyruvate formate lyase family (see, for example, [56] for an overview of the reaction mechanism). A similar mechanism of activation, i.e. addition to fumarate, was proposed for the anaerobic degradation of alkanes based on the finding of alkylsuccinates in cultures of sulfate-reducing bacteria growing with *n*-dodecane [57], and of a nitrate-reducing bacterium, ‘*Aromatoleum*’ sp. strain HxN1 growing on *n*-hexane [58]. Following these initial observations, activation by addition to fumarate has been demonstrated for the degradation of a relatively broad range of *n*-alkanes (C_6_–C_16_) and cycloalkanes by nitrate- and sulfate-reducing cultures (e.g. [49,59–62]). In the case of alkanes, the most common site of activation is the subterminal (secondary) carbon atom, yielding (1-methylalkyl)succinates, although metabolites were identified indicating also an activation at the C-3 atom [58], and at the terminal (C-1) carbon atom [46]. The formed alkylsuccinates have been proposed to be further degraded by ligation to coenzyme A, carbon-skeleton re-arrangement and beta-oxidation [46,63]. This yields chiefly acetyl-CoA which is subjected to terminal oxidation to CO_2_. In sulfate-reducing bacteria, the most common pathway for the oxidation of acetyl-CoA is the Wood-Ljungdahl pathway. The presence of this pathway in alkane degraders has been demonstrated by measurements of the central enzyme, carbon monoxide dehydrogenase, in the hexadecane degrading strain Hxd3 [64], and by genome sequencing of the *n*-alkane degrading *Desulfatibacillum alkenivorans* AK-01 [65].

An alternative mechanism of activation via subterminal carboxylation at the C-3 atom of the alkane has been suggested based on the analysis of fatty acids and metabolites formed during growth of the sulfate-reducing bacterium *Desulfococcus oleovorans* Hxd3 on long-chain *n*-alkanes [66–68]. Recent biochemical investigations indicated that alkane activation in *D. oleovorans* Hxd3 actually takes place via hydroxylation followed by carboxylation [69]. In addition, a third mechanism of activation with the possible involvement of a N—O species or O_2_, generated intracellularly from the electron acceptor, has been proposed for the *Gammaproteobacterium* strain HdN1 [70].

**Fig. 2:**
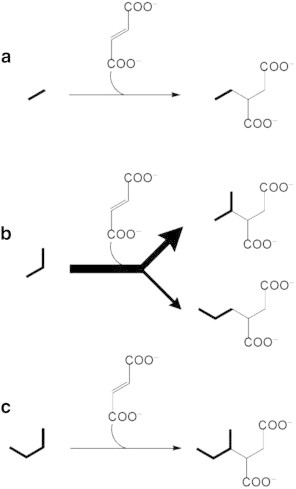
Proven and proposed mechanisms for the biochemical activation of gaseous alkanes under anoxic conditions, by addition to fumarate yielding alkylsuccinates. For ethane, activation by addition to fumarate (a) can be envisioned based on the finding of propane activation at a terminal carbon atom, and by the identification of ethylsuccinate in environmental samples. Propane is activated by addition to fumarate at both the subterminal and terminal carbon atoms (b), yielding isopropyl- and *n*-propylsuccinate, respectively, with a higher frequency of activation events at the secondary carbon atom. Butane is activated only at the subterminal carbon atom (c), yielding (1-methylpropylsuccinate).

Up to now, most investigations on the mechanism of alkane activation under anoxic conditions have been focused on the degradation of medium and long-chain *n*-alkanes (> C_6_) [49]. With respect to gaseous alkanes, relatively recent metabolite analyses have shown that activation by addition to fumarate also plays an important role. In cultures of strain BuS5 and of propane-degrading enrichment cultures, isopropylsuccinate and (1-methylpropyl)succinate were identified as metabolites, indicating that propane and *n*-butane are activated by addition to fumarate at the secondary carbon atom (Fig. 2) [46,48]. In addition, *n*-propylsuccinate was identified in propane-degrading cultures, suggesting a second route of propane activation at the primary carbon atoms [46,48]. Although initially considered as a side reaction, this second pathway was further substantiated by incubations of strain BuS5 with position-specific deuterium-labeled propane. It was shown that the activation of propane at the primary carbon atoms is significant, accounting for an estimated 30% of the activation events, with the bulk of 70% of activation events occurring at the secondary carbon atom [71]. The activation of propane at the primary carbon atoms opens up the possibility of ethane activation by a similar mechanism, yielding ethylsuccinate (Fig. 2). Although up to now no metabolites have been reported from anaerobic ethane-degrading cultures, this hypothesis is supported by the finding of ethylsuccinate in crude oil processing facilities, crude oil production wells [42], oilfields [40], and coal beds [41].

The analogy of alkane activation by addition to fumarate to the anaerobic activation of alkyl-aromatic hydrocarbons (e.g. toluene) led to the identification of genes and proteins similar with the benzylsuccinate synthase (Bss) in anaerobic alkane-utilizing microorganisms. Based on similarity with subunits of the Bss, genes encoding for alkane-activating glycyl-radical enzymes have been identified in the sulfate-reducing bacterium *D. alkenivorans* AK-01 [72], and in the nitrate-reducing strain ‘*Aromatoleum*’ sp. HxN1 [73], and termed alkylsuccinate synthase (Ass) [72] and (1-methylalkyl)succinate synthase (Mas) [73], respectively. The putative large, catalytic subunit of these enzymes (AssA or MasD) harbors a conserved radical-hosting Gly residue within a motif characteristic for the Gly-radical enzymes [56]. In ‘*Aromatoleum*’ sp. HxN1, transcripts and proteins of several *mas* genes were detected in cells grown on *n*-hexane, but were absent in cells grown on caproate [73]. These included MasD, with a high sequence identity to the large (α) subunit of the Bss, MasC, a small protein similar to the γ subunit of the Bss, and MasE, a protein rich in Cys residues, typical of the β subunit of the Bss. Based on their specific expression in *n*-hexane-grown cells, and similarities with subunits of the benzylsuccinate synthase, MasC, MasD and MasE were interpreted as subunits of the (1-methylalkyl)succinate synthase in strain HxN1 [73]. In *D. alkenivorans* AK-01, two loci coding for the proposed alkylsuccinate synthase have been identified based on homology to subunits of Mas and Bss [65,72]. Each of these loci contains genes encoding the putative catalytic subunit (AssA1/AssA2), and putative β and γ subunits (AssB1/2, AssC1/2) [65]. In addition, the two loci contain genes similar with the *masE* of ‘*Aromatoleum*’ sp. HxN1 [65]. However, only one of the putative α subunits (AssA1) was found to be expressed in cells grown on hexadecane and not on hexadecanoate; the role of AssA2 is presently unknown [65]. Subsequently, proteins similar to Ass/Mas have been also detected in other model alkane-utilizing strains such as *Desulfoglaeba alkanexedens* ALDC [74], and ‘*Aromatoleum*’ sp. OcN1 [70]. Recent genomic analyses led to the identification of *assA*-like genes in *Smithella* spp. from methanogenic enrichment cultures with *n*-hexadecane [75,76], and with *n*-alkanes > C_6_ [76,77], and in a *Firmicutes* sp. (*Peptococcaceae* SCADC) from a methanogenic enrichment culture with *n*-alkanes > C_6_ [78] (Fig. 2). These findings indicate that alkane activation in methanogenic cultures may also proceed via addition to fumarate.

**Fig. 3:**
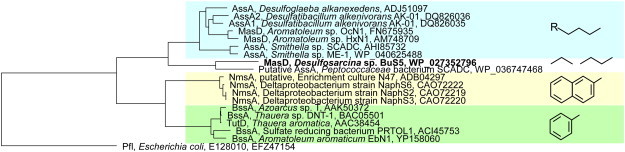
Phylogenetic reconstruction showing the relationship of the putative MasD of strain BuS5 (in boldface), likely involved in propane and *n*-butane activation, with other *n*-alkane and alkylaromatic hydrocarbon activating enzymes. R ≥ C_2_. The tree was calculated in ARB by maximum likelihood, using the pyruvate-formate lyase of *E. coli* as an outgroup. F. Musat and S. Sievert, unpublished.

Identification of methylalkylsuccinate synthase genes of gaseous alkane degrading microorganisms is still incipient. A partial gene sequence similar with *assA* was retrieved from a mesophilic, sulfate-reducing enrichment culture with propane [48,74]. Recent genome analysis of strain BuS5 identified a single putative *masD* gene, suggesting that strain BuS5 contains a single methylalkylsuccinate synthase involved in the activation of both propane and *n*-butane (F. Musat and S. Sievert, unpublished). Phylogenetic analysis of the putative MasD showed that it is clustering with other glycyl-radical enzymes involved in hydrocarbon activation, with a higher similarity to other methylalkylsuccinate synthases (Fig. 3). The putative MasD of strain BuS5 apparently forms a branch together with the putative AssA from the *Peptococcaceae* sp. SCADC separate from other MasD proteins (Fig. 3). Nevertheless, the two proteins share about 60% sequence identity (F. Musat and S. Sievert, unpublished), and it remains for future studies to establish if the Mas/Ass from gaseous alkane degraders indeed form a separate lineage vs. similar proteins from medium- and long-chain alkane degraders. In view of the anticipated diversity of SCA degraders indicated by recent studies, a high diversity of corresponding masD/assA genes is expected to be uncovered from anoxic environments around natural gas and crude oil seeps.

## 6 Conclusion

Evidence for the anaerobic degradation of gaseous alkanes has been provided for various subsurface environments, mostly marine sediments from hydrocarbon seepage areas. From such environments, the first microorganisms able to degrade short-chain alkanes under anoxic conditions have been enriched or isolated. Both microbiological and geochemical studies suggest that propane and *n*-butane appear more susceptible to biodegradation than ethane and *iso*-butane, despite the relatively high abundance of the latter in natural gas. Analysis of metabolites demonstrated that under anoxic conditions propane and *n*-butane are biochemically activated by addition to fumarate at the secondary carbon atom, a mechanism similar to the activation of higher *n*-alkanes, cycloalkanes and alkyl-aromatic hydrocarbons. For propane, an additional route of activation at the primary carbon atoms has been shown, which could in principle be employed also for the activation of ethane or *iso*-butane.

So far, most of the anaerobic gaseous alkane degraders cultivated or otherwise identified by molecular biology methods are sulfate-reducing bacteria which belong to the *Desulfosarcina*–*Desulfococcus* cluster of the *Deltaproteobacteria*. Members of this group are generally abundant in anoxic marine sediments, and in recent studies have been directly linked to biodegradation of SCA in environmental samples. These findings point out to a major, if not global contribution to the marine carbon and sulfur cycles. Quantitatively, a larger fraction of the natural gas in marine environments is released through microseepage than through the more conspicuous gas seeps. It would be indeed interesting for future studies to determine if gaseous alkane degraders have a more widespread distribution in anoxic marine sediments than presently recognized.
